# Immune architecture of colorectal cancer brain metastases: spatial TAM heterogeneity and PD-L1 dynamics

**DOI:** 10.3389/fimmu.2026.1816086

**Published:** 2026-04-13

**Authors:** Lisa Marie Grape, Benjamin Hanke, Belal Neyazi, Ali Rashidi, Vanessa Magdalena Swiatek, Anna Schaufler, Claudia Alexandra Dumitru, Ulf Kahlert, Roland Croner, Christian Mawrin, Sabine Franke, I. Erol Sandalcioglu, Klaus-Peter Stein

**Affiliations:** 1 Department of Neurosurgery, Otto-von-Guericke University, Magdeburg, Germany; 2 Institute of Pathology, Otto-von-Guericke University, Magdeburg, Germany; 3 Institute of Pathology, Medizinisches Versorgungszentrum (MVZ) Städtisches Klinikum Dessau, Magdeburg, Germany; 4 Department of General, Visceral, Vascular and Transplant Surgery, Otto-von-Guericke University, Magdeburg, Germany; 5 Institute of Neuropathology, Otto-von-Guericke University, Magdeburg, Germany

**Keywords:** brain metastases, colorectal cancer, PD-L1, TAMs, tumor microenvironment, tumor-associated macrophages

## Abstract

**Background:**

Brain metastases from colorectal cancer (CRC) are associated with poor survival and limited treatment options. As immunomodulatory therapies gain relevance, a deeper understanding of the tumor microenvironment (TME) in this setting is needed. Tumor-associated macrophages (TAMs) are pivotal regulators of tumor immunity, yet their spatial organization, polarization, and relationship to PD-L1–mediated immune checkpoint regulation in CRC brain metastases remain poorly defined. We therefore characterized the compartment-specific architecture and functional orientation of TAMs in brain metastases and matched primary CRCs.

**Methods:**

Immunohistochemical analyses of CD68 (pan-macrophages), CD86 (M1-associated), CD163 (M2-associated), and PD-L1 were performed on tissue microarrays from tumor specimens of 50 patients with CRC brain metastases, including 31 matched primary tumor–brain metastasis pairs. Compartment-specific TAM densities and PD-L1 expression were quantified to assess intra- and intertumoral heterogeneity and correlated with clinicopathological parameters and clinical outcomes.

**Results:**

Both primary CRCs and brain metastases exhibited macrophage-rich TMEs characterized by stromal predominance and an M2-skewed polarization. Compared with matched primary tumors, brain metastases showed a significant stromal enrichment of CD163^+^ TAMs. Dexamethasone treatment was associated with reduced densities of CD86^+^ TAMs in brain metastases. PD-L1 expression was predominantly confined to immune cells and displayed marked intra- and intertumoral heterogeneity, with frequent discordance between matched primary tumors and brain metastases, including recurrent lesions. In primary CRCs, high densities of CD68^+^ TAMs at the invasive front were associated with shortened brain metastasis–free survival, whereas neither TAM infiltration nor PD-L1 expression correlated with overall survival.

**Conclusion:**

CRC brain metastases exhibit a distinct, stroma-dominated and M2-polarized TME, consistent with site-specific enrichment of protumoral TAM phenotypes within the cerebral niche. This may reflect advanced disease biology, immunological adaptation to the brain microenvironment, or therapy- and selection-driven immune remodeling during metastatic progression. The association between dexamethasone treatment and reduced M1-associated TAM infiltration suggests therapy-related modulation of antitumoral immune activity, with potential implications for perioperative management. The heterogeneity and frequent discordance of PD-L1 expression highlight its dynamic regulation and support individualized assessment of metastatic lesions prior to immunotherapy. Collectively, these findings support site-specific immune profiling and identify TAMs as promising therapeutic targets within the TME of CRC brain metastases.

## 1 Introduction

Colorectal cancer (CRC) is one of the most common malignancies worldwide and a leading cause of cancer-related mortality, largely driven by the development of distant metastases, with 5-year survival rates below 20% (1, 2). Brain metastases are rare, occurring in approximately 1–2% of patients, yet they are associated with limited therapeutic options and a dismal median survival of 3–7 months (3–5). Their incidence appears to be increasing, likely reflecting improved systemic disease control and prolonged patient survival (6). Despite their clinical relevance, the biological and immunological determinants of CRC brain metastases remain poorly understood.

Tumor progression and metastatic outgrowth are critically shaped by the tumor microenvironment (TME), a complex network of stromal components, extracellular matrix, and immune cells (7, 8). In CRC, heterogeneity in immune composition contributes to variable disease behavior and therapeutic response (9, 10). Among immune cells, tumor-associated macrophages (TAMs) represent one of the most abundant and functionally versatile populations within the TME (11, 12). Macrophages display pronounced plasticity and adopt a broad spectrum of activation states in response to external cues, commonly conceptualized along an M1/M2 axis. M1 macrophages are characterized by expression of co-stimulatory molecules such as CD86 and exert proinflammatory and antitumoral functions through antigen presentation and cytokine release. In contrast, M2 macrophages express scavenger receptors including CD163 and promote tissue remodeling, angiogenesis, immunosuppression, and tumor progression (13, 14). In most solid tumors, increased TAM infiltration — particularly with M2-like features — is associated with adverse clinical outcomes (15–17).

Beyond polarization, TAM function is highly context dependent and varies across distinct tumor regions (18). At the invasive front, TAMs facilitate matrix remodeling, suppress cytotoxic immune responses, and promote epithelial–mesenchymal transition (19–21), while perivascular stromal niches support angiogenesis and tumor cell intravasation (22–25). Hypoxic tumor regions further reinforce immunosuppressive programs, highlighting the importance of spatially resolved analyses (26).

The unique cerebral microenvironment imposes additional, tissue-specific constraints on immune regulation. Brain metastasis formation is accompanied by blood–brain barrier disruption, recruitment of peripheral monocytes, and reprogramming of resident microglia toward TAM-like phenotypes in response to local cytokine and metabolic cues (27–29). Accumulating evidence suggests that these conditions favor immunosuppressive macrophage programs, which may be co-opted by metastatic tumor cells to foster a tumor-promoting niche within the brain (30–32).

A key immunoregulatory pathway linking TAMs to immune evasion is the PD-1/PD-L1 checkpoint axis (33, 34). While immune checkpoint blockade has shown durable efficacy in a subset of microsatellite instability–high CRCs, most tumors — particularly microsatellite-stable CRCs — remain largely resistant (35). TAMs constitute a major source of PD-L1 within the TME and critically modulate checkpoint signaling in both tumor and immune cells (33, 34). In brain metastases, PD-L1 expression by TAMs and microglia contributes to a markedly immunosuppressive milieu and may limit therapeutic efficacy (30, 32).

Despite increasing understanding of TAM biology and immune checkpoint pathways, the spatial immune architecture of CRC brain metastases — particularly TAM polarization and its association with PD-L1 expression — remains poorly defined. Accordingly, this study aimed to systematically analyze the compartment-specific distribution and functional orientation of TAMs in CRC brain metastases and matched primary tumors using immunohistochemical profiling. Integration of spatial immune features with clinical outcome data provides a framework for understanding site-specific immune regulation and for exploring microenvironment-directed therapeutic targets.

## 2 Materials and methods

### 2.1 Study design and patient cohort

This retrospective, single-center study included patients with histologically confirmed CRC brain metastases who underwent neurosurgical resection between 2007 and 2022. All analyses were performed on formalin-fixed, paraffin-embedded (FFPE) tumor tissue. The study was approved by the local ethics committee (No. 146/19) and conducted in accordance with the Declaration of Helsinki. Inclusion criteria comprised availability of sufficient viable tumor tissue, confirmed colorectal origin of the brain metastasis, and access to clinicopathological and follow-up data. A total of 50 patients with CRC brain metastases were included, of whom 31 had matched primary CRC and brain metastasis specimens available for paired analyses. Primary tumor location was classified based on ICD-O-3 coding. For rectal cancers, anatomical localization was further stratified into upper, middle, and lower thirds according to UICC definitions. Clinicopathological parameters and survival data were retrieved from medical records.

### 2.2 Tissue microarray construction and spatial compartment definition

Representative FFPE blocks of brain metastases and available matched primary CRC specimens were selected based on hematoxylin and eosin–stained sections. Tissue microarrays (TMAs) were constructed to enable standardized immunohistochemical analyses across all cases. Each FFPE block was assessed for predefined intratumoral compartments, including tumor nests, tumor stroma, and – where identifiable – the invasive front. Representative 3 mm core biopsies were obtained from each compartment and assembled into TMA recipient blocks. This approach allowed spatially resolved assessment of TAM infiltration while minimizing inter-assay variability. To mitigate potential sampling bias inherent to TMA-based analyses, relatively large core diameters were used and tumor compartments were predefined based on histomorphological criteria. In addition, multiple representative hotspot regions were evaluated per compartment, enabling robust assessment of spatial immune heterogeneity.

### 2.3 Immunohistochemistry

Immunohistochemical staining was performed on 2 µm TMA sections using antibodies against CD68 (pan-macrophages), CD86 (M1-associated), CD163 (M2-associated), and PD-L1. Staining was performed using validated protocols with appropriate positive and negative controls. PD-L1 and DNA mismatch repair (MMR) protein expression (MLH1, PMS2, MSH2, MSH6) were assessed using automated staining platforms according to established diagnostic standards. Detailed antibody specifications and staining protocols are provided in the **Supplementary Methods** (**Supplementary Table S1**).

### 2.4 Evaluation of TAM infiltration and PD-L1 expression

**Figure 1:**
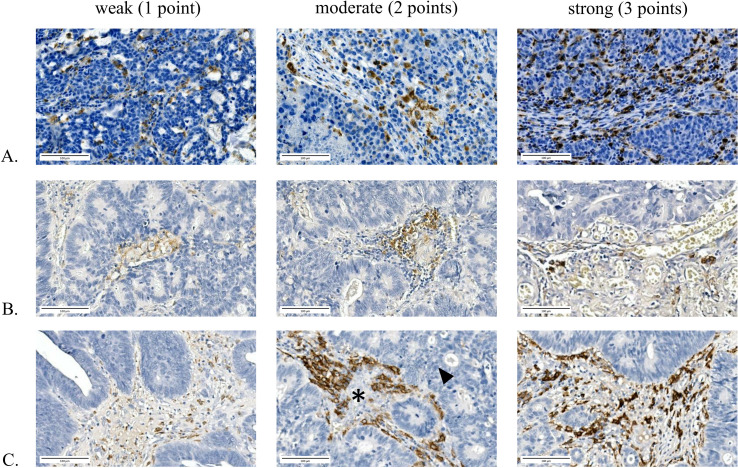
TAM intensity scoring. TAM intensity scores were determined based on the staining intensity of immunohistochemical labeling. From left to right, representative examples of weak, moderate, and strong staining of CD68, CD86, and CD163 in both primary CRCs and brain metastases are shown, with corresponding score assignment. In the middle image of **(C)**, tumor nests are indicated by an arrowhead, whereas the surrounding macrophage-rich tumor stroma is marked by (*). **(A)** CD68 staining intensity. **(B)** CD86 staining intensity. **(C)** CD163 staining intensity. All images are shown at 20× digital magnification.

**Figure 2:**
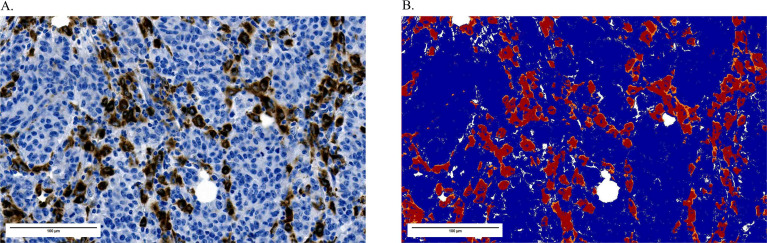
TAM reactivity scoring. **(A)** Representative immunohistochemical staining showing strong CD68^+^ TAM reactivity (brown) in a brain metastasis in the presence of negative tumor nests (blue). **(B)** Corresponding quantitative assessment using the Positive Pixel Count algorithm. Brown pixels are classified as positive and displayed in red, pixels of other hues appear blue, and unstained pixels are shown in white. The resulting reactivity score was 17%. All images are shown at 20× digital magnification.

Immunostained slides were digitally scanned and evaluated using Aperio ImageScope software (Leica Biosystems). TAMs were identified based on marker-specific staining patterns in conjunction with cellular morphology. For each tumor compartment, up to five representative hotspot regions were selected at 20× magnification. TAM infiltration was assessed using a modified semi-quantitative immunoreactive score (IRS), calculated as the product of staining intensity and the proportion of positive cells, with adjusted percentage thresholds to better capture differences in macrophage density across tumor compartments (**Figure 1**). In cases with borderline reactivity scores, quantification was supported by the Aperio Positive Pixel Count algorithm (Leica Biosystems) to ensure standardized score assignment (**Figure 2**). PD-L1 expression was evaluated separately in tumor and immune cells. A combined positive score (CPS) was calculated, and PD-L1 positivity was defined as CPS ≥ 1, in accordance with previous studies.

### 2.5 Clinical endpoints

Brain metastasis–free survival was defined as the interval from initial CRC diagnosis to the diagnosis of brain metastasis. Overall survival (OS) was defined as the interval from the initial diagnosis of colorectal cancer to death or last follow-up. In cases in which brain metastases were diagnosed prior to the primary colorectal tumor, and for analyses correlating immune marker expression in brain metastases with outcome, OS was calculated from the date of brain metastasis diagnosis.

### 2.6 Statistical analysis

Statistical analyses were performed using R (R Foundation for Statistical Computing, v4.3.0). Differences in TAM densities between tumor compartments and between primary tumors and brain metastases were analyzed using non-parametric tests. For association analyses with clinicopathological parameters and for survival analyses, IRS values of CD68, CD86, and CD163 were dichotomized into high and low expression groups using median-based cut-offs. Associations with clinicopathological variables were assessed using Fisher’s exact test or regression models as appropriate. Survival analyses were conducted using the Kaplan-Meier method and log-rank tests. A two-sided p-value ≤ 0.05 was considered statistically significant.

## 3 Results

### 3.1 Patient characteristics

The study cohort comprised 50 patients with histologically confirmed CRC brain metastases who underwent neurosurgical resection. Thirty-two patients (64%) were male and 18 (36%) female. Age at initial CRC diagnosis ranged from 40 to 84 years, with a median age of 64 years, while the median age at diagnosis of brain metastasis was 66 years. Primary tumor location was classified as right-sided colon cancer (RCC) in 16 patients (32%), left-sided colon cancer including upper third rectal primaries (LCC) in 10 patients (20%), and rectal cancer involving the middle and lower thirds in 24 patients (48%).

Brain metastases were detected synchronously with or prior to the primary tumor in seven patients, whereas the remaining patients developed metachronous brain metastases after a median interval of 28.5 months. Median OS from initial CRC diagnosis was 41 months, while median OS from the time of brain metastasis diagnosis was 9.5 months. No significant differences in OS were observed between anatomical subgroups (log-rank test, p > 0.05). Cohort characteristics are summarized in **Table 1**. Detailed clinicopathological characteristics are provided in **Supplementary Table S2**.

**Table 1:** Clinical characteristics of the study cohort.

Characteristic	Total cohort (n = 50)
Age at CRC diagnosis, median (IQR)	64 (58–70.5)
Age at BM diagnosis, median (IQR)	66 (59.75–75.25)
Sex, n (%)	
– Male	32 (64)
– Female	18 (36)
Primary tumor location, n (%)	
– Right-sided colon	16 (32)
– Left-sided colon	10 (20)
– Rectum	24 (48)
BM diagnosis, n (%)	
– Synchronous/prior to primary CRC	7 (14)
– Metachronous	43 (86)
Number of BMs, n (%)	
– Solitary	34 (68)
– Multiple	16 (32)
Time to BM, months, median (IQR)	28.5 (12.5–56)
OS from CRC diagnosis, months, median (IQR)	41 (24.5–69)
OS from BM diagnosis, months, median (IQR)	9.5 (5–12.5)

CRC, colorectal cancer; BM, brain metastasis; IQR, interquartile range; OS, overall survival.

### 3.2 Spatial distribution of tumor-associated macrophages

To characterize the spatial immune architecture of CRC brain metastases, TAMs were analyzed across predefined intratumoral compartments, including tumor nests, tumor stroma, and the invasive front. Expression of CD68 (pan-macrophages), CD86 (M1-associated), and CD163 (M2-associated) was assessed using a compartment-specific modified IRS.

TAMs were detected across all compartments in both primary CRCs and brain metastases, with significant enrichment in the stromal compartment compared with tumor cell nests across all markers (Wilcoxon signed-rank tests for paired comparisons; all p < 0.001; **Table 2**). In primary CRCs, TAM densities at the invasive front were also significantly higher than within tumor nests, particularly for CD68 and CD163.

**Table 2:** Intratumoral distribution of TAMs across tumor compartments.

A) Primary CRCs					
Marker	Compartment	Median IRS (IQR)	p (vs TN)	p (vs TS)	p (vs IF)
CD68	TN	2 (0–2)	–	**< 0.001**	**< 0.001**
	TS	3 (3–6)	**< 0.001**	–	0.79
	IF	3 (2–6)	**< 0.001**	0.79	–
CD86	TN	0 (0–0)	–	**< 0.001**	**< 0.01**
	TS	2 (0–3)	**< 0.001**	–	0.09
	IF	1.5 (0–3)	**< 0.01**	0.09	–
CD163	TN	0 (0–0)	–	**< 0.001**	**< 0.001**
	TS	3 (1–4)	**< 0.001**	–	0.25
	IF	4 (2–6)	**< 0.001**	0.25	–
B) Brain metastases					
Marker	Compartment	Median IRS (IQR)		p (TN vs TS)	
CD68	TN	2 (0–2)		**< 0.001**	
	TS	3 (2–6)			
CD86	TN	0 (0–0)		**< 0.001**	
	TS	1 (0–2.25)			
CD163	TN	0 (0–1)		**< 0.001**	
	TS	6 (2–9)			

IRS, immunoreactive score; IQR, interquartile range; TN, tumor nests; TS, tumor stroma; IF, invasive front.

A) Median IRS (IQR) per compartment is shown. p values from paired Wilcoxon signed-rank tests: p (vs TN), p (vs TS) and p (vs IF) indicate the comparison tested (n = 31, IF evaluable in n = 27 due to tissue exhaustion).

B) Median IRS (IQR) per compartment is shown. p values indicate TN vs TS comparisons (paired Wilcoxon signed-rank test, n = 50).

Bold values represent statistically significant p-values.

These findings were confirmed by cumulative link mixed models, which demonstrated significantly lower IRS values in tumor nests relative to other compartments in both primary tumors and brain metastases (all p < 0.001). Across compartments, CD86 expression was consistently lower than CD68 and CD163 expression. Detailed results and subgroup analyses by primary tumor location are shown in **Supplementary Table S3**.

### 3.3 Primary tumors versus brain metastases

To assess site-specific differences in TAM infiltration, paired analyses were performed in the subset of 31 patients with available matched primary CRC and brain metastasis specimens. Overall, TAM densities were comparable between primary tumors and metastases; however, compartment-specific differences in macrophage marker expression were observed.

CRC brain metastases exhibited significantly higher densities of CD163^+^ TAMs in the tumor stroma compared with matched primary tumors (Wilcoxon signed-rank test; p = 0.048). This difference was confined to the stromal compartment and was not observed for CD68 or CD86 expression. CD86^+^ TAM densities remained low and did not differ significantly between primary tumors and metastases across compartments (**Table 3**).

**Table 3:** Paired comparison of TAM densities between primary CRCs and brain metastases.

Marker	Compartment	CRC, median IRS (IQR)	BM, median IRS (IQR)	p value
CD68	Tumor stroma	3 (3–6)	3 (2–6)	0.515
CD86	Tumor stroma	2 (0–3)	1 (0–2.25)	0.189
CD163	Tumor stroma	3 (1–4)	6 (2–9)	**0.048**

IRS, immunoreactive score; IQR, interquartile range; CRC, colorectal cancer; BM, brain metastasis.

p values were calculated using Wilcoxon signed-rank tests for paired intertumoral comparisons (n = 31 pairs). Only TS is shown, as no significant paired differences were observed in TN or IF.

Bold values represent statistically significant p-values.

Subgroup analyses stratified by primary tumor location (RCC, LCC, rectum) did not reveal statistically significant differences. A trend toward higher stromal CD163 expression in brain metastases arising from rectal primaries was observed but did not reach statistical significance (p ≈ 0.07). Complete paired analyses across all compartments are provided in **Supplementary Table S4**.

### 3.4 Prognostic relevance of TAMs

The prognostic relevance of macrophage infiltration was evaluated separately for primary CRCs and brain metastases. In primary tumors, TAM abundance was correlated with brain metastasis–free survival, defined as the interval from CRC diagnosis to the diagnosis of brain metastasis.

**Figure 3:**
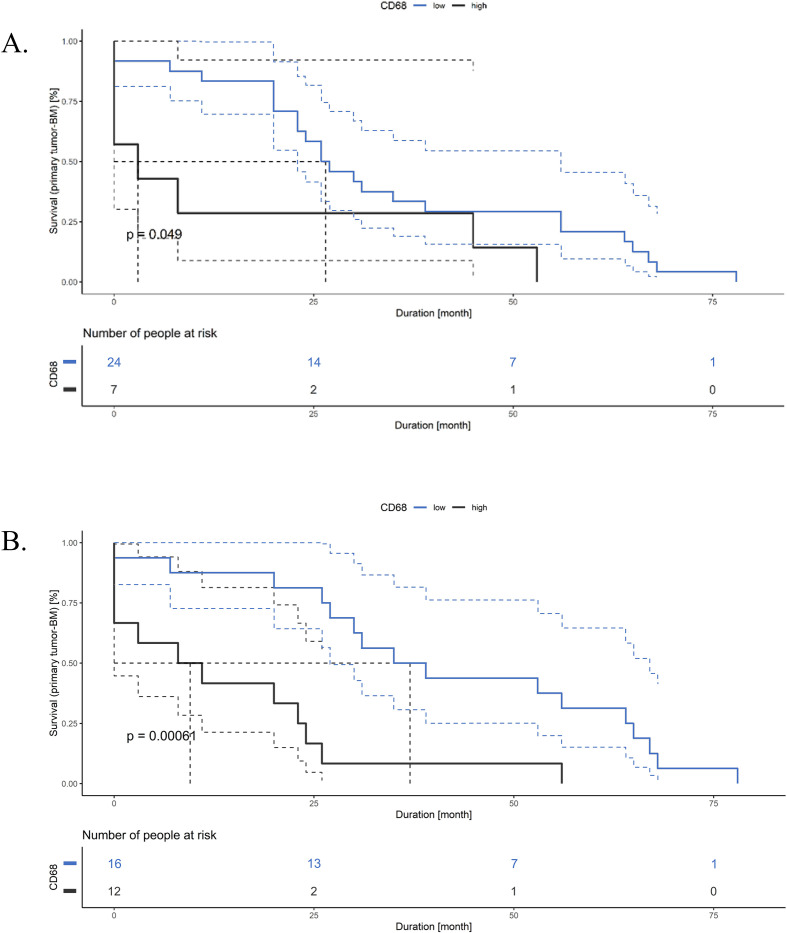
Impact of CD68 expression on brain metastasis–free survival. Kaplan-Meier curves illustrating brain metastasis–free survival stratified by CD68 expression in different regions of the primary tumor, with p-values derived from the log-rank test. **(A)** Impact of CD68 expression in tumor nests of primary CRCs. **(B)** Impact of CD68 expression at the invasive front of primary CRCs.

High densities of CD68^+^ TAMs within tumor nests and at the invasive front of primary CRCs were associated with a significantly shorter brain metastasis–free survival (**Table 4**). Median brain metastasis–free survival was 27 months for CD68-low versus 3 months for CD68-high tumors in tumor nests (log-rank tests; p = 0.049). At the invasive front, median brain metastasis–free survival was 37 months for CD68-low versus 9.5 months for CD68-high tumors (p < 0.001). Corresponding Kaplan-Meier curves are shown in **Figure 3**. No comparable associations were observed for CD86 or CD163 expression in other compartments.

**Table 4:** Association of TAM infiltration with survival outcomes.

Marker	Compartment	n (low vs high)	Median BFS (low vs high), months	p value
CD68	Tumor nest	13 vs 18	27 vs 3	**0.049**
CD68	Invasive front	16 vs 12	37 vs 9.5	**< 0.001**

BFS, brain metastasis–free survival.

Group assignment (low vs high) by median IRS split; p values from log-rank tests (n = 31, IF evaluable in n = 28 due to tissue exhaustion).

Bold values represent statistically significant p-values.

In contrast, TAM infiltration within brain metastases did not correlate with overall survival calculated from the time of brain metastasis diagnosis. Analysis of overall survival from initial CRC diagnosis likewise revealed no significant associations with TAM abundance in primary tumors. The results are shown in **Supplementary Table S5**.

### 3.5 Associations with clinicopathological parameters

Associations between TAMs and clinicopathological parameters were examined for both primary CRCs and brain metastases. In primary tumors, high stromal CD68 expression was more frequently observed in patients with synchronous brain metastases compared with those with metachronous disease (Fisher’s exact test; p = 0.012). CD86 expression in primary CRCs showed no consistent associations with clinicopathological parameters, with only isolated near-significant findings. In contrast, CD163 expression in primary tumors was associated with selected pathological features. Lower CD163 expression within tumor nests was observed in low-grade tumors (p = 0.043) and in adenocarcinomas, NOS (p = 0.038). The presence of distant metastases at initial diagnosis was associated with lower stromal CD163 expression (p = 0.046).

In brain metastases, prior dexamethasone treatment was associated with significantly lower stromal CD86 expression (p = 0.002). Absence of cerebral recurrence correlated with lower CD163 expression within tumor nests (p = 0.033). No further robust associations with clinicopathological variables were identified. Detailed results are provided in **Supplementary Table S6**.

### 3.6 PD-L1 expression and intertumoral discordance

PD-L1 expression was assessed in primary CRCs and brain metastases using the combined positive score (CPS), incorporating both tumor and immune cell staining. PD-L1 positivity (CPS ≥ 1) was detected in 16 of 31 primary tumors (52%) and in 18 of 49 brain metastases (37%), with marked intertumoral heterogeneity.

In both primary CRCs and brain metastases, PD-L1 expression was predominantly observed in immune cells, whereas tumor cell PD-L1 expression was infrequent. All CPS-positive tumors exhibited PD-L1 expression in immune cells, whereas tumor cell PD-L1 expression was detected in only a minority of CPS-positive cases (4/16 [25%] primary tumors; 5/18 [28%] brain metastases). Cumulative link mixed models demonstrated a significant association between PD-L1 positivity and higher TAM densities (**Supplementary Table S3**).

**Figure 4:**
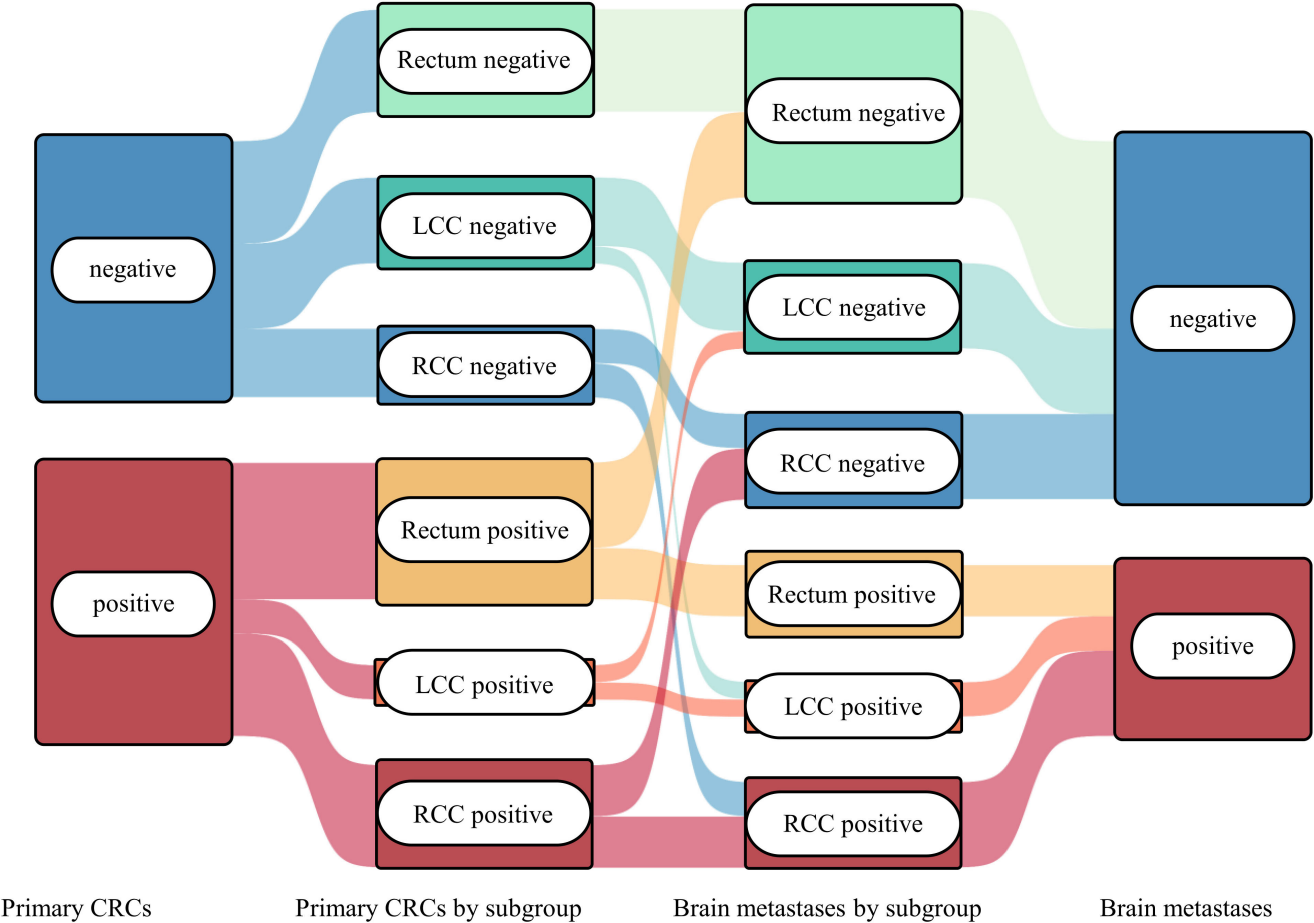
Comparison of PD-L1 status between primary tumors and metastases. Sankey diagram depicting concordance and discordance of PD-L1 status between primary tumors and corresponding brain metastases, stratified by anatomical subgroups (RCC, LCC, and rectum).

Paired analysis of matched primary tumors and brain metastases revealed limited concordance of PD-L1 expression between sites (**Figure 4**). Discordant PD-L1 status was observed in 11 of 30 evaluable pairs (3 cases PD-L1^+^ in metastasis only, 8 cases PD-L1^+^ in primary only). Accordingly, Fisher’s exact test showed no significant predictive association (p = 0.245). Among eight additionally analyzed recurrent or newly occurring brain metastases, seven were PD-L1 positive. Five of these cases exhibited newly acquired PD-L1 positivity compared with the corresponding initial tumors, while loss of PD-L1 expression was not observed.

PD-L1 expression was neither associated with brain metastasis–free survival or OS, irrespective of whether survival was calculated from CRC diagnosis or from the diagnosis of brain metastasis. Detailed results are included in **Supplementary Table S5**.

**Figure 5:**
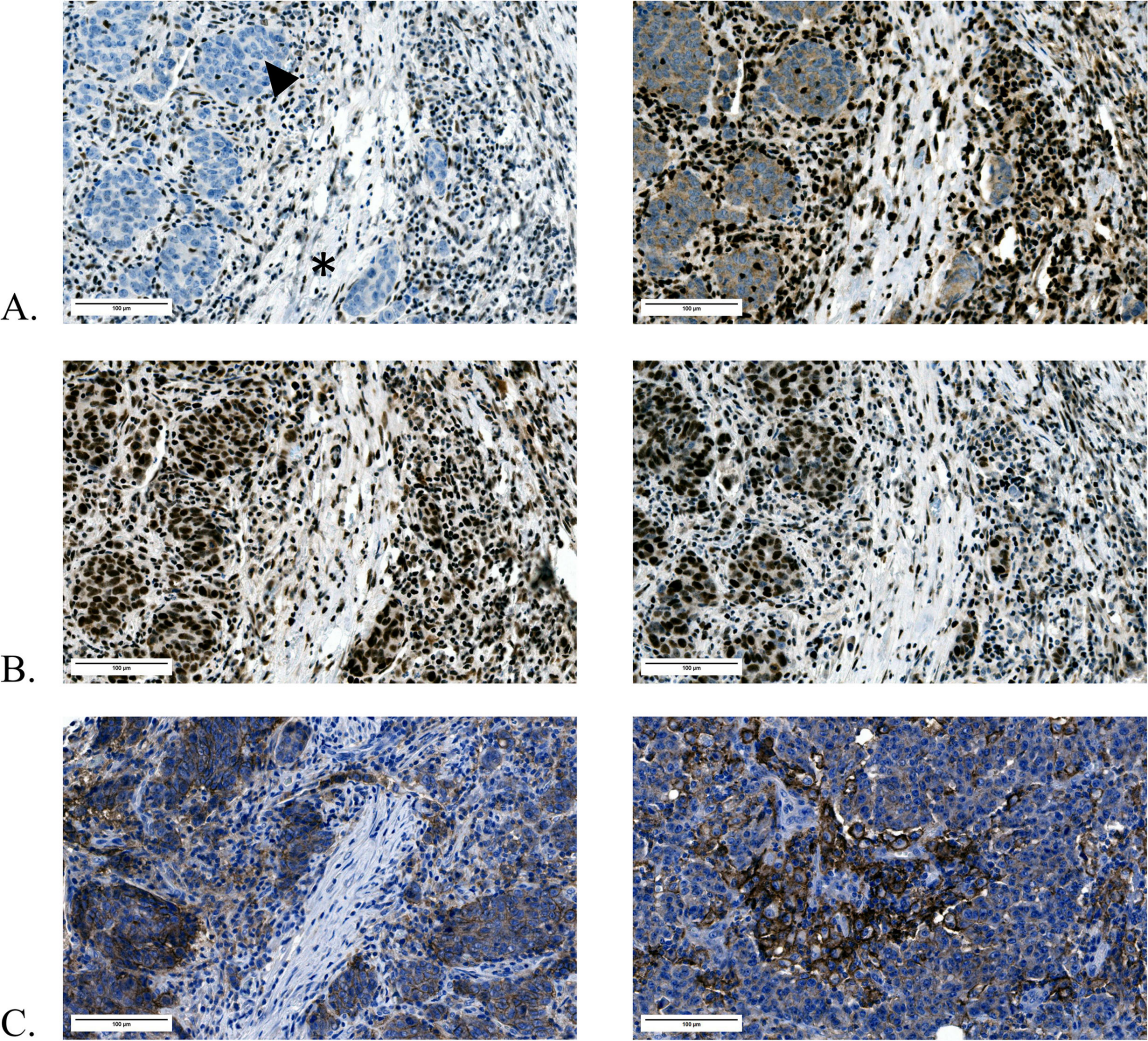
Association of MMR deficiency and PD-L1 expression. Classical immunohistochemical pattern of MMR protein loss and PD-L1 expression in an MMR-deficient primary CRC. **(A)** Dual loss of MLH1 (left) and PMS2 (right) expression in tumor cells (arrowhead) with preserved staining in stromal cells (*) serving as internal positive controls. **(B)** Preserved nuclear expression of MSH2 (left) and MSH6 (right) in tumor cells with intact internal controls. **(C)** Corresponding PD-L1 expression in the same tumor, demonstrating strong membranous staining in tumor cells (left) and in immune cells, particularly macrophages (right). A comparable expression pattern was observed in the corresponding brain metastasis (not shown). All images are shown at 15× magnification.

MMR protein expression was assessed in 23 primary tumors and all 50 brain metastases due to its established relevance for immune checkpoint therapy. MMR deficiency was identified in two primary CRCs (9%), both showing loss of MLH1 and PMS2 expression. In both cases, MMR status was concordant between primary tumors and corresponding brain metastases and was associated with high PD-L1 expression (CPS up to 95), as shown in **Figure 5**. In addition, one brain metastasis from a patient without available primary tumor tissue exhibited MMR deficiency. All rectal cancers were MMR proficient. Detailed MMR data are provided in **Supplementary Table S2**.

## 4 Discussion

### 4.1 Principal findings

In this study, we provide a comprehensive spatial and intertumoral analysis of TAMs and PD-L1 expression in CRC brain metastases and matched primary tumors. By integrating compartment-resolved immunohistochemistry with paired primary-metastasis analyses and clinical outcome data, we demonstrate that CRC brain metastases are embedded in a distinct immune microenvironment. Our data reveal (1) a pronounced stromal dominance of TAMs with an overall M2-skewed polarization in both primary CRCs and brain metastases; (2) a site-specific enrichment of CD163^+^ TAMs within the stromal compartment of brain metastases compared with matched primary tumors; (3) a significant association between preoperative dexamethasone treatment and reduced infiltration of CD86^+^ TAMs in brain metastases; and (4) marked intertumoral heterogeneity of PD-L1 expression, predominantly driven by immune cells and frequently discordant between primary and metastatic lesions.

Together, these observations establish the brain metastatic niche as a distinct immunological compartment, in part shaped by macrophage-mediated immunosuppression and dynamic regulation of immune checkpoint signaling.

### 4.2 Spatial organization and polarization of TAMs

Our spatial analyses demonstrate that TAM infiltration in CRC is highly compartment dependent, with consistent enrichment in stromal regions and, in primary tumors, at the invasive front. Rather than reflecting uniform immune infiltration, this pattern suggests preferential recruitment and retention of macrophages at tumor–host interfaces where immune regulation, invasion, and stromal remodeling converge.

Across all compartments, CD163^+^ macrophages markedly outnumbered the uniformly scarce CD86^+^ population, indicating a generally immunosuppressive macrophage landscape in metastatic CRC. This observation is consistent with previous reports describing stromal and perivascular enrichment of M2-like TAMs, potentially mediated by EGF signaling in CRC (36–39). In contrast, M1-associated signatures are generally less pronounced and tend to be confined to peritumoral regions (39, 40). This compartmentalization may therefore amplify local immunosuppression while shielding tumor cell nests from direct immune surveillance.

These results emphasize that bulk quantification of macrophage density may obscure biologically relevant spatial differences. Spatially resolved analyses are therefore essential to better understand the functional heterogeneity of TAMs within the TME.

### 4.3 Site-specific immune reprogramming in the brain metastatic niche

A central finding of this study is the selective enrichment of CD163^+^ TAMs within the stromal compartment of CRC brain metastases compared with matched primary tumors. Importantly, this shift was not accompanied by a global increase in macrophage density or by changes in CD86 expression, indicating qualitative immune reprogramming rather than quantitative immune cell expansion.

Several, non-mutually exclusive mechanisms may account for this M2 enrichment in brain metastases. First, it may reflect advanced disease biology, as brain metastases typically occur late in the metastatic cascade of CRCs and are often associated with prolonged and aggressive systemic disease courses (41, 42). In this context, accumulation of M2-polarized TAMs may represent a cumulative endpoint of chronic immunosuppression accompanying metastatic progression.

Second, the cerebral metastatic niche imposes unique immunological constraints that may actively shape macrophage polarization. Our data support a model in which CRC cells entering the brain encounter a microenvironment that favors the induction or stabilization of immunosuppressive macrophage programs, likely through interactions with disseminated tumor cells, resident cell populations, altered metabolic conditions, and cytokine gradients emerging after blood–brain barrier disruption. The stromal confinement of this CD163 enrichment suggests that immune reprogramming occurs preferentially at tumor–host interfaces, where direct crosstalk between tumor cells, stromal elements, and immune cells is most pronounced, rather than within tumor cell nests.

Third, therapy- and selection-driven immune remodeling may contribute to the observed enrichment of M2-associated TAMs. During the metastatic disease course, tumor cells are often exposed to various treatment regimens, including chemotherapy, radiotherapy, and immunotherapy, all of which can exert selective pressure on both tumor and immune compartments (43, 44). Tumor cell clones capable of co-opting immunosuppressive macrophage programs may therefore be preferentially selected during metastatic outgrowth, particularly within the tightly regulated immune environment of the brain.

Consistent with this interpretation, similar patterns of macrophage-mediated immunosuppression have been reported in brain metastases from breast cancer and non-small cell lung cancer, suggesting that M2-dominated macrophage landscapes may represent a conserved feature of the cerebral metastatic niche rather than a CRC-specific phenomenon. While Lui et al. describe a more immunosuppressive cerebral TME, Zimmer et al. reported higher TAM densities in brain metastases compared with their matched primary tumors (45, 46). In this context, TAMs may facilitate immune evasion by limiting cytotoxic T cell activity and sustaining an immunosuppressive niche that shields tumor cells and supports metastatic outgrowth and persistence.

Collectively, these findings indicate that CRC brain metastases are not merely immunological extensions of the primary tumor but represent a site-specific immune microenvironment shaped by disease progression, local microenvironmental cues, and therapeutic selection. This has important implications for immunotherapeutic strategies, as approaches effective in extracranial disease may require adaptation to address macrophage-driven immunosuppression within the brain.

### 4.4 Prognostic implications of TAMs

The prognostic relevance of TAMs differed markedly between primary CRCs and established brain metastases, indicating context-dependent functions of the macrophage compartment. In primary tumors, high densities of CD68^+^ TAMs within tumor nests and at the invasive front were associated with a significantly shorter brain–metastasis free survival, whereas no prognostic associations were observed within the metastatic lesions themselves. Zimmer et al. reported similar results, with high CD163 expression associated with shorter brain metastasis–free survival but not with OS (45). Interestingly, high stromal CD68 expression was also more frequently observed in patients with synchronous brain metastases.

This divergence suggests that TAMs may primarily contribute to metastatic initiation rather than determining outcome once brain metastases are established. In the primary tumor, macrophage-rich niches at the invasive front and within tumor cell nests may foster early dissemination by promoting extracellular matrix remodeling, immune evasion, tumor cell intravasation, and the establishment of pre-metastatic niches. This, in turn, may enable tumor cells to survive in distant, highly immune-regulated sites such as the brain.

In contrast, the absence of a prognostic impact of TAM infiltration in brain metastases suggests that, at this disease stage, clinical outcome is likely dominated by factors such as overall tumor burden, neurological complications, and treatment-related constraints, potentially outweighing microenvironmental influences detectable by immunohistochemistry. However, Liu et al. reported an association between TAM infiltration and poor outcomes in lung cancer brain metastases, and Friebel et al. similarly observed shorter OS associated with CD163^+^ TAMs in brain metastases (46, 47).

These findings may help reconcile conflicting reports on the prognostic value of TAMs in CRC. Previous studies have linked macrophage infiltration to both favorable and adverse outcomes, likely reflecting differences in tumor stage, macrophage subtypes, spatial localization, and methodological approaches (48, 49). Our data underscore that prognostic interpretations of macrophage-associated markers must consider spatial context and disease stage to avoid oversimplification.

### 4.5 PD-L1 expression and intertumoral discordance

PD-L1 expression in both primary CRCs and brain metastases was predominantly confined to immune cells and closely associated with macrophage-rich tumor regions, whereas tumor cell PD-L1 expression was infrequent. This pattern is consistent with prior observations indicating that M2-polarized TAMs represent a major source of PD-L1 in CRC, particularly at the invasive front (50–53). Furthermore, TAMs modulate immunotherapy responsiveness through multiple mechanisms, including sequestration of therapeutic anti–PD-1 antibodies from T cells in CRC (54). This supports a model in which immune checkpoint regulation in CRC is partly orchestrated by the macrophage compartment rather than tumor cells themselves, reinforcing the central role of TAMs in shaping local immunosuppression.

Notably, PD-L1 expression showed limited concordance between matched primary tumors and brain metastases. Discordant PD-L1 status was observed in a substantial proportion of paired cases, with a tendency toward newly acquired PD-L1 positivity in recurrent or progressive brain lesions. These observations are consistent with previous studies reporting frequent discordance in PD-L1 expression between primary and metastatic CRC lesions, with a tendency toward higher expression in metastases (55, 56). This highlights the dynamic nature of immune escape mechanisms during disease progression and suggests that PD-L1 expression may be shaped by site-specific immune pressures rather than being a static tumor-intrinsic feature. Previous studies have reported variable, in some cases high, PD-L1 expression in brain metastases from other tumor entities, accompanied by heterogeneous efficacy of immune checkpoint inhibitor therapy (57–60).

Despite its biological relevance, PD-L1 expression did not correlate with survival outcomes, consistent with its limited prognostic value in CRC (61). Nevertheless, the observed spatial and temporal heterogeneity and frequent discordance argue against sole reliance on primary tumor tissue for immunotherapeutic decision-making and support the need for site-specific immune profiling in the metastatic setting.

### 4.6 MMR status

MMR deficiency was infrequent in our cohort but consistently associated with high PD-L1 expression. Concordance of MMR status between primary tumors and brain metastases suggests genetic stability of this biomarker during metastatic spread. Notably, all rectal cancers in our cohort were MMR proficient, consistent with epidemiological data (62). While the small number of MMR-deficient cases precludes definitive conclusions, these findings align with the established immunogenic phenotype of MMR-deficient CRC and support continued integration of MMR status into immune profiling strategies in metastatic CRC.

### 4.7 Clinical and therapeutic implications

The immune architecture identified in this study has several translational implications. First, pronounced stromal enrichment of M2-polarized macrophages indicates that immune suppression in the cerebral metastatic niche is not solely driven by tumor cells but is in part orchestrated by the macrophage compartment. This may, at least in part, explain the limited efficacy of immune checkpoint inhibition in this setting and indicates that macrophage-mediated immune regulation represents a critical therapeutic barrier. This suggests that therapeutic strategies targeting TAM abundance, polarization, or function may represent a rational complement to immune checkpoint inhibition in CRC brain metastases, where current immunotherapeutic efficacy remains variable.

Second, the observed association between preoperative dexamethasone exposure and reduced infiltration of M1-associated TAMs suggests therapy-related modulation of antitumoral immune activity within brain metastases. While corticosteroids are often indispensable for perioperative management of cerebral edema, these findings raise the possibility that steroid-induced immune modulation may inadvertently reinforce an immunosuppressive microenvironment. Accordingly, recent studies across brain metastases and glioblastoma have consistently linked greater dexamethasone exposure to worse survival and potentially reduced benefit from immunotherapy (63–65). This observation warrants further investigation and may have implications for optimizing perioperative management strategies in patients with CRC brain metastases, particularly in the context of immunotherapeutic approaches targeting the PD-1/PD-L1 axis.

Third, the frequent discordance of PD-L1 expression between primary tumors and brain metastases further underscores the limitations of biomarker assessment based solely on primary tumor tissue. Whenever feasible, immune profiling of metastatic lesions may provide more accurate guidance for immunotherapeutic interventions.

Finally, the association between TAM-rich primary tumors and early brain metastatic progression suggests that macrophage-targeted therapies could be explored not only in the treatment of established metastases but also in strategies aimed at prolonging metastasis–free survival.

### 4.8 Strengths and limitations

Strengths of this study include the use of matched primary-metastatic tumor pairs, spatially resolved immune profiling across predefined tumor compartments, and integration with clinical outcome data. However, several limitations should be acknowledged. The retrospective design and limited cohort size constrain statistical power, particularly for subgroup analyses. Moreover, immunohistochemistry represents a surrogate approach and cannot fully capture the functional and transcriptional heterogeneity of macrophages. In particular, classification of TAMs based on CD68, CD86, and CD163 reflects a simplified approximation of macrophage polarization along the M1/M2 axis and does not account for the full spectrum of activation states within the tumor microenvironment. While combined marker panels may improve phenotypic characterization, they remain limited in resolving the functional complexity of TAM populations.

Larger, prospective studies integrating spatial transcriptomics or multiplex imaging will be required to validate and extend these findings.

## 5 Conclusion

In this study, we demonstrate that CRC brain metastases are characterized by a distinct TME with pronounced stromal accumulation of M2-associated TAMs. Spatial analyses reveal marked compartment- and site-specific differences in TAM distribution and functional orientation between primary tumors and brain metastases, underscoring the importance of individual immune profiling.

In primary CRCs, TAM infiltration was associated with earlier development of brain metastases, suggesting a role in metastatic initiation, whereas selective stromal enrichment of M2-associated TAMs in brain metastases indicates immune reprogramming within the cerebral niche that may promote immunosuppression and metastatic outgrowth. Preoperative dexamethasone treatment was associated with reduced densities of M1-associated TAMs, pointing to therapy-related modulation of antitumoral immune activity. Frequent discordance between PD-L1 expression in primary CRCs and brain metastases highlights its dynamic regulation during disease progression and supports the assessment of metastatic tissue prior to immunotherapy.

Together, these findings identify TAMs as central components of the immune architecture of CRC brain metastases and support macrophage-directed therapeutic strategies. Integrating macrophage-targeted approaches with established systemic and local treatments may help overcome site-specific immune resistance and improve outcomes in this challenging disease context.

## Data Availability

The raw data supporting the conclusions of this article will be made available by the authors, without undue reservation.
