# Supplementary material for: Immune architecture of colorectal cancer brain metastases: spatial TAM heterogeneity and PD-L1 dynamics
Source: Front Immunol. 2026 Apr 13;17:1816086. doi: 10.3389/fimmu.2026.1816086 (PMC13111462; doi:10.3389/fimmu.2026.1816086)
Supplement: Supplementary file 1 [file DataSheet1.pdf]

Supplementary Material

1 Supplementary Methods

Tissue microarray construction

Tissue microarrays (TMAs) were constructed from formalin-fixed, paraffin-embedded (FFPE) tumor blocks using an Arraymold Kit. Representative tumor regions were selected based on hematoxylin and eosin–stained sections to ensure sufficient tumor content and accurate sampling of the desired tumor compartment. Core biopsies with a diameter of 3 mm were obtained from predefined tumor compartments, including tumor nests, tumor stroma, and — where applicable — the invasive front, and assembled into recipient paraffin blocks.

Immunohistochemistry

Immunohistochemical staining was performed on FFPE TMA sections following standardized protocols. Staining for CD68 and PD-L1 was performed on automated platforms according to routine diagnostic standards, whereas CD86 and CD163 were stained manually using established protocols.

Mismatch repair (MMR) protein expression (MLH1, PMS2, MSH2, and MSH6) was assessed by immunohistochemistry using validated diagnostic antibodies. Nuclear staining in tumor cells was evaluated according to the manufacturer's interpretation guidelines, with loss of expression of at least one MMR protein interpreted as MMR deficiency in the presence of intact internal controls.

Antigen retrieval was performed using heat-induced epitope retrieval. Endogenous peroxidase activity was blocked, followed by incubation with primary antibodies and detection using polymer-based detection systems. All staining runs included appropriate positive and negative controls.

Detailed antibody specifications, including clone, manufacturer, and dilution, are provided in Supplementary Table S1.

Table S1. Antibody specifications.

CD68	clone KP1	Cell Marque, Rocklin, CA, US	1:500
CD86	clone E2G8P	Cell Signaling Technology, Danvers, MA, US	1:250
CD163	clone QA19A16	BioLegend, San Diego, CA, US	1:2000
PD-L1	clone 22C3	Agilent Technologies, Santa Clara, CA, US	1:50
MLH1	clone M1	Roche, Basel, Switzerland	ready to use
PMS2	clone A16-4	Roche, Basel, Switzerland	ready to use
MSH2	clone G219-1129	Cell Marque, Rocklin, CA, US	1:200
MSH6	clone 44	Cell Marque, Rocklin, CA, US	1:100

Evaluation of tumor-associated macrophages

All slides were scanned using a high-resolution digital slide scanner. Image analysis was performed using Aperio ImageScope software. Tumor-associated macrophages (TAMs) were identified based on marker-specific staining patterns and cellular morphology. For each tumor compartment, representative hotspot regions were selected for analysis.

<u>Modified immunoreactive score</u>

TAM infiltration was assessed using a modified semi-quantitative immunoreactive score (IRS). Staining intensity was scored as follows: 0 (no staining), 1 (weak), 2 (moderate), or 3 (strong). The proportion of positive cells (reactivity score) was scored using adapted percentage thresholds: 0 (0%), 1 (1–10%), 2 (11–20%), 3 (21–40%), and 4 (> 40%).

These percentage thresholds were adapted from previously published IRS systems to allow improved discrimination of macrophage densities, particularly in compartments with lower overall immune cell infiltration. The final IRS was calculated as the product of the intensity score and the reactivity score.

<u>Handling of borderline reactivity scores</u>

In cases with borderline reactivity scores close to predefined category thresholds, digital image analysis was performed using the Aperio Positive Pixel Count algorithm (Leica Biosystems). The algorithm quantified the proportion of positively stained pixels within selected regions of interest at high magnification.

Results from the Positive Pixel Count analysis were used to support assignment of the reactivity score by providing an objective measure of staining extent. This approach was applied uniformly across tumor compartments to minimize observer-dependent variability and did not replace observer-based assessment.

PD-L1 assessment

PD-L1 expression was evaluated separately in tumor cells and immune cells. A combined positive score (CPS) was calculated according to established criteria, and PD-L1 positivity was defined as CPS ≥ 1, in line with published clinical studies in gastrointestinal malignancies.

2 Supplementary Tables

Table S2. Extended clinicopathological characteristics.

A) Primary CRCs

		RCC		LCC		Rectum	
		**n**	**%**	**n**	**%**	**n**	**%**
**Total**		10	100	7	100	14	100
**Gender**	male	6	60	6	85.7	10	71.4
	female	4	40	1	14.3	4	28.6
**Age at diagnosis**	≤ 64 years	4	40	3	42.9	10	71.4
	> 64 years	6	60	4	57.1	4	28.6
**Invasion depth (T)**	2	1	10	0	0	1	7.1
	3	7	70	5	71.4	12	85.7
	4	2	20	2	28.6	1	7.1
**Lymph node metastasis (N)**	N0	2	20	2	28.6	3	21.4
	N+	8	80	5	71.4	11	78.6
**Distant metastasis (M)**	M0	5	50	3	42.9	11	78.6
	M1	5	50	4	57.1	3	21.4
**Histological subtype**	NOS	7	70	7	100	12	85.7
	M/SR	3	30	0	0	2	14.3
**Grading**	low	5	50	4	57.1	13	92.9
	high	5	50	3	42.9	1	7.1
**MMR status**	pMMR	9	90	3	42.9	10	71.4
	dMMR	1	10	1	14.3	0	0
	indeterminate	0	0	3	42.9	4	28.6
**Neoadjuvant therapy**	yes	1	10	1	14.3	10	71.4
	no	9	90	6	85.7	4	28.6

NOS = not otherwise specified, M/SR = mucinous / signet ring cell component

B) Brain metastasis

		RCC		LCC		Rectum	
		**n**	**%**	**n**	**%**	**n**	**%**
**Total**		16	100	10	100	24	100
**Gender**	male	10	62.5	8	80	14	58.3
	female	6	37.5	2	20	10	41.7
**Occurrence**	synchronous	3	18.8	1	10	3	12.5
	metachronous	13	81.3	9	90	21	87.5
**Age at diagnosis**	≤ 66 years	7	43.8	4	40	16	66.7
	> 66 years	9	56.3	6	60	8	33.3
**Number of brain metastases**	solitary	11	68.8	5	50	18	75
	multiple	5	31.3	5	50	6	25
**Dexamethasone treatment**	yes	8	50	5	50	13	54.2
	no	5	31.3	4	40	5	20.8
	indeterminate	3	18.8	1	10	6	25
**Relapse or new metastasis**	yes	8	50	3	30	10	41.7
	no	8	50	7	70	14	58.3
**MMR status**	pMMR	14	87.5	9	90	24	100
	dMMR	2	12.5	1	10	0	0

Table S3. Intratumoral TAM distribution by subgroup and association with PD-L1 expression.

A) Wilcoxon signed-rank tests

	Primary CRC							Brain metastasis			
	**n**	**Median IRS**			**p**			**n**	**Median IRS**		**p**
		**TN**	**TS**	**IF**	**TN-TS**	**TN-IF**	**TS-IF**		**TN**	**TS**	
**CD68**											
Total	31	2	3	3	**0.000025*** **	**0.000008*** **	0.791793	50	2	3	**0.000001*** **
RCC	10	2	5	3	0.014059*	0.016438*	1.000000	16	2	3	0.007000**
LCC	7	2	3	3	0.067889	0.025597*	0.853923	10	2	4	0.027000*
Rectum	14	0	3	5	0.002081**	0.001918**	0.738883	24	1	3	0.000413***
**CD86**											
Total	31	0	2	1,5	**0.000045*** **	**0.001218** **	0,090693	50	0	1	**0.000005*** **
RCC	10	0	2	0	0.010909*	0.067889	0.496242	16	0	1	0.010000*
LCC	7	0	3	2	0.025597*	0.038434*	0.157299	10	0	2	0.014000*
Rectum	14	0	2	1	0.015764*	0.063318	0.285049	24	0	0	0.002968**
**CD163**											
Total	31	0	3	4	**0.000002*** **	**0.000003*** **	0.252904	50	0	6	**< 0.000001*** **
RCC	10	0	2	4	0.006606**	0.007526**	0.144127	16	0	3	0.001000**
LCC	7	0	3	2	0.017350*	0.015959*	0.407626	10	1	6	0.012000*
Rectum	14	0	3	5	0.000937***	0.002015**	0.450351	24	0	6	0.000025***

TN = tumor nest, TS = tumor stroma, IF = invasion front; *p ≤ 0.05, **p ≤ 0.01, ***p ≤ 0.001

B) Cumulative link mixed models

	Primary CRC			Brain metastasis		
	**log(OR)**	**95% CI**	**p**	**log(OR)**	**95% CI**	**p**
**CD86**	-2.0	-2.7; -1.3	**< 0.001**	-3.1	-3.8; -2.4	**< 0.001**
**Tumor nest**	-3.8	-4.6; -3.1	**< 0.001**	-2.5	-3.5; -1.5	**< 0.001**
**Positive PD-L1 status**	1.2	0.24; 2.2	**0.014**	1.3	0.59; 2.1	**< 0.001**

OR, Odds Ratio (higher IRS), CD86 vs CD68 and CD163, TN vs TS and IF; CI = Confidence Interval.

Table S4. Wilcoxon signed-rank test results for intertumoral comparisons of TAM distribution.

	Tumor nest				Tumor stroma			
	**n**	**Median IRS CRC**	**Median IRS BM**	**p**	**n**	**Median IRS CRC**	**Median IRS BM**	**p**
**CD68**								
Total	31	2	2	0.304	31	3	3	0.515
RCC	10	2	2	0.301	10	4	3	0.472
LCC	7	2	2	0.890	7	3	3	1.000
Rectum	14	0	0	0.764	14	3	3	0.622
**CD86**								
Total	31	0	0	0.784	31	2	1	0.189
RCC	10	0	0	1.000	10	2	2	0.837
LCC	7	0	0	1.000	7	3	2	0.457
Rectum	14	0	0	0.371	14	1	0	0.259
**CD163**								
Total	31	0	0	0.683	31	3	6	**0.048***
RCC	10	0	0	0.586	10	2	2	0.443
LCC	7	0	0	1.000	7	3	4	0.529
Rectum	14	0	0	1.000	14	3	6	0.073

Table S5. Association of TAMs and PD-L1 expression with survival outcomes.

	Brain metastasis-free survival				OAS (CRC)				OAS (BM)			
	**Median**	**n**	**n***	**p**	**Median**	**n**	**n***	**p**	**Median**	**n**	**n***	**p**
**TN**												
CD68-low	27 (23, 56)	31	31	**0.049**	40 (35, 68)	31	30	0.2	10 (8.0, 12)	50	43	0.9
CD68-high	3,0 (0.00, —)				25 (16, —)				11 (5.0, —)			
CD86-low	26 (20, 45)	31	31	0.8	40 (34, 60)	31	30	0.6	10 (8.0, 12)	50	43	0.8
CD86-high	8.0 (3.0, —)				25 (14, —)				12 (4.0, —)			
CD163-low	26 (20, 56)	31	31	0.3	38 (34, 72)	31	30	0.2	8.0 (6.0, 13)	50	43	0.7
CD163-high	22 (3.0, —)				35 (23, —)				12 (11, 17)			
**TS**												
CD68-low	23 (20, 45)	31	31	0.3	42 (30, 72)	31	30	> 0.9	9.0 (7.0, 12)	50	43	0.6
CD68-high	28 (23, 68)				36 (34, 88)				12 (9.0, 23)			
CD86-low	24 (20, 45)	31	31	0.8	36 (30, 73)	31	30	> 0.9	10 (8.0, 16)	50	43	0.4
CD86-high	35 (8.0, —)				58 (42, —)				10 (7.0, 16)			
CD163-low	23 (20, 56)	31	31	0.9	36 (30, 73)	31	30	0.6	10 (8.0, 16)	50	43	0.4
CD163-high	29 (8.0, —)				42 (25, —)				11 (7.0, —)			
**IF**												
CD68-low	37 (27, 67)	28	**28**	**< 0.001**	51 (38, 73)	28	28	0.2	—	—	—	—
CD68-high	9,5 (0.00, —)				30 (23, —)				—			
CD86-low	29 (26, 68)	28	28	0.2	42 (36, 88)	28	28	0.2	—	—	—	—
CD86-high	22 (8.0, 64)				36 (30, 72)				—			
CD163-low	29 (20, 64)	29	29	0.2	40 (30, 73)	29	29	0.4	—	—	—	—
CD163-high	23 (3.0, —)				36 (30, —)				—			
**PD-L1 status**												
Negative	35 (20, 65)	31	31	0.13	44 (27, 74)	31	30	0.6	9.0 (7.0, 13)	49	42	0.8
Positive	23 (3.0, 45)				36 (30, 72)				12 (10, 23)			

Median in months with 95% CI provided in parentheses; OAS (CRC), calculated from CRC diagnosis; OAS (BM), calculated from brain metastasis diagnosis; n*, n (event); TN = tumor nest, TS = tumor stroma, IF = invasion front; —, not applicable.

Table S6. Associations between TAMs and clinicopathological parameters.

A) CD68 (Primary CRCs)

	CD68, TN			CD68, TS			CD68, IF		
	**high, n = 7**	**low, n = 24**	**p**	**high, n = 14**	**low, n = 17**	**p**	**high, n = 12**	**low, n = 16**	**p**
**Invasion depth (T)**			0.7			0.4			> 0.9
2	1	1		2	0		1	1	
3	5	19		10	14		9	13	
4	1	4		2	3		2	2	
**Lymph node metastasis (N)**			0.6			0.2			> 0.9
N0	2	5		5	2		3	4	
N+	5	19		9	15		9	12	
**Distant metastasis (M)**			0.4			0.5			0.4
M0	3	16		10	9		6	11	
M1	4	8		4	8		6	5	
**Histological subtype**			0.6			> 0.9			0.6
NOS	5	21		12	14		11	13	
M/SR	2	3		2	3		1	3	
**Grading**			0.4			0.13			0.1
Low	4	18		12	10		7	14	
High	3	6		2	7		5	2	
**Neoadjuvant therapy**			0.4			0.5			> 0.9
Yes	4	8		4	8		4	5	
No	3	16		10	9		8	11	
**BM occurrence**			> 0.9			**0.012**			0.13
Synchronous	1	4		5	0		4	1	
Metachronous	6	20		9	17		8	15	

TN = tumor nest, TS = tumor stroma, IF = invasion front; NOS = not otherwise specified, M/SR = mucinous / signet ring cell component; BM, brain metastasis.

B) CD86 (Primary CRCs)

	CD86, TN			CD86, TS			CD86, IF		
	**high, n = 3**	**low, n = 28**	**p**	**high, n = 11**	**low, n = 20**	**p**	**high, n = 14**	**low, n = 14**	**p**
**Invasion depth (T)**			0.5			> 0.9			**0.048**
2	0	2		1	1		0	2	
3	2	22		8	16		10	12	
4	1	4		2	3		4	0	
**Lymph node metastasis (N)**			0.12			0.2			> 0.9
N0	2	5		4	3		4	3	
N+	1	23		7	17		10	11	
**Distant metastasis (M)**			0.3			0.5			> 0.9
M0	3	16		8	11		8	9	
M1	0	12		3	9		6	5	
**Histological subtype**			> 0.9			0.3			> 0.9
NOS	3	23		8	18		12	12	
M/SR	0	5		3	2		2	2	
**Grading**			0.2			0.2			0.7
Low	1	21		5	16		9	11	
High	2	7		6	4		5	3	
**Neoadjuvant therapy**			0.5			0.5			0.4
Yes	1	8		3	6		3	6	
No	2	20		8	14		11	8	
**BM occurrence**			0.4			0.6			> 0.9
Synchronous	1	4		1	4		2	3	
Metachronous	2	24		10	16		12	11	

TN = tumor nest, TS = tumor stroma, IF = invasion front; NOS = not otherwise specified, M/SR = mucinous / signet ring cell component; BM, brain metastasis.

C) CD163 (Primary CRCs)

	CD163, TN			CD163, TS			CD163, IF		
	**high, n = 6**	**low, n = 25**	**p**	**high, n = 10**	**low, n = 21**	**p**	**high, n = 11**	**low, n = 18**	**p**
**Invasion depth (T)**			0.5			> 0.9			0.8
2	0	2		1	1		1	1	
3	4	20		8	16		8	15	
4	2	3		1	4		2	2	
**Lymph node metastasis (N)**			0.6			0.2			> 0.9
N0	2	5		4	3		3	4	
N+	4	20		6	18		8	14	
**Distant metastasis (M)**			> 0.9			**0.046**			0.7
M0	4	15		9	10		7	10	
M1	2	10		1	11		4	8	
**Histological subtype**			**0.038**			> 0.9			0.3
NOS	3	23		8	18		8	16	
M/SR	3	2		2	3		3	2	
**Grading**			**0.043**			0.7			0.4
Low	2	20		8	14		7	14	
High	4	5		2	7		4	4	
**Neoadjuvant therapy**			> 0.9			0.7			> 0.9
Yes	2	10		3	9		4	6	
No	4	15		7	12		7	12	
**BM occurrence**			> 0.9			> 0.9			> 0.9
Synchronous	1	4		2	3		2	3	
Metachronous	5	21		8	18		9	15	

TN = tumor nest, TS = tumor stroma, IF = invasion front; NOS = not otherwise specified, M/SR = mucinous / signet ring cell component; BM, brain metastasis.

D) CD68 (Brain metastasis)

	CD68, TN			CD68, TS		
	**high, n = 11**	**low, n = 39**	**p**	**high, n = 17**	**low, n = 33**	**p**
**Number of BMs**			0.7			0.8
Solitary	7	27		11	23	
Multiple	4	12		6	10	
**Dexamethasone**			0.1			0.7
Yes	3	23		9	17	
No	5	9		6	8	
Indeterminate	3	7		2	8	
**Relapse, new BM**			> 0.9			> 0.9
Yes	5	16		7	14	
No	6	23		10	19	

TN = tumor nest, TS = tumor stroma; BM, brain metastasis.

E) CD86 and CD163 (Brain metastasis)

	CD86, TN			CD86, TS			CD163, TN			CD163, TS		
	**high, n = 4**	**low, n = 46**	**p**	**high, n = 23**	**low, n = 27**	**p**	**high, n = 17**	**low, n = 33**	**p**	**high, n = 15**	**low, n = 35**	p
**Number of BMs**			0.6			0.14			0.8			0.2
Solitary	2	32		13	21		11	23		8	26	
Multiple	2	14		10	6		6	10		7	9	
**Dexamethasone**			0.11			**0.002**			> 0.9			> 0.9
Yes	1	25		8	18		9	17		8	18	
No	3	11		12	2		4	10		4	10	
Indeterminate	0	10		3	7		4	6		3	7	
**Relapse, new BM**			0.6			0.6			**0.033**			0.5
Yes	1	20		11	10		11	10		5	16	
No	3	26		12	17		6	23		10	19	

TN = tumor nest, TS = tumor stroma; BM, brain metastasis.
